# Hypertriglyceridaemia and its multifaceted mechanisms driving atherosclerotic cardiovascular disease​​

**DOI:** 10.1186/s12944-026-02983-6

**Published:** 2026-06-04

**Authors:** Xintong Li, Lishuang Xu, Jingwei Li, Yong Luo, Hang Shen, Daqing Zhang

**Affiliations:** Cardiology Department, Shengjing Hospital of China Medical University, Shenyang, China

**Keywords:** Hypertriglyceridaemia, Atherosclerosis, Lipoprotein metabolism, Cholesterol accumulation, Inflammatory reaction

## Abstract

Emerging evidence has shown that hypertriglyceridaemia (HTG) is a significant independent residual risk factor for atherosclerotic cardiovascular disease (ASCVD), even with optimal low density lipoprotein cholesterol (LDL-C) management. Although its direct causal role in atherogenesis remains debatable, recent mechanistic insights have unveiled novel proatherogenic pathways associated with HTG. This review critically synthesises and updates the evolving evidence linking HTG to atherosclerotic pathogenesis. Here, we describe multiple mechanisms by which HTG contributes to the initiation and progression of atherosclerotic plaques. These include promotion of subendothelial cholesterol retention, induction of vascular inflammation and endothelial dysfunction, acceleration of arterial aging, and promotion of prothrombosis. Integrating these contemporary insights, this review outlines a refined scientific framework that posits that HTG is a crucial modifiable residual risk factor for ASCVD. This paradigm enables more comprehensive risk stratification and informs the development of targeted therapies to complement conventional lipid-lowering treatments.

**Supplementary Information:**

The online version contains supplementary material available at 10.1186/s12944-026-02983-6.

## Introduction

Atherosclerotic cardiovascular disease (ASCVD) remains a major cause of death and disability [1]. Over recent decades, statin therapy has substantially reduced low-density lipoprotein cholesterol (LDL-C) and major adverse cardiovascular events (MACE) in patients with moderate to very high cardiovascular risk. Multiple landmark trials from the 1990 s and 2000s—such as WOSCOPS, 4S, CARE, and JUPITER—have consistently demonstrated that statin therapy can lower major cardiovascular events by roughly one-quarter to one-third. However, even with this benefit, approximately 70% of the baseline cardiovascular risk remains unaddressed [2–7]. More intensive statin regimens (PROVE IT–TIMI 22, TNT, IDEAL [8], TNT [9], and IDEAL [10]) achieved additional relative risk reductions of 16%, 22%, and 11%, yet significant residual risks of 22.4%, 8.7%, and 12% persisted, highlighting the limits of statin monotherapy. Adding ezetimibe or PCSK9 inhibitors can lower LDL-C to historically low levels (< 1.4 mmol/L) and further reduce ASCVD risk [11–14]. Nevertheless, a considerable residual risk remains. For example, the long-term (8.4-year) extension of the FOURIER trial reported a median LDL-C of 0.78 mmol/L with evolocumab plus statin but only a 15% relative risk reduction [12]. These findings indicate that even profoundly low LDL-C levels do not fully eliminate cardiovascular risk, underscoring the need to address non-LDL drivers of ASCVD.

Several trails (EMPATHY [15], MIRACL [16], PROVE IT-TIMI 22 [17], and dal-OUTCOMES [18]) have shown that lower triglyceride (TG) levels independently predict fewer adverse cardiovascular events in patients on intensive statin therapy. This underscores that triglyceride-rich lipoproteins (TRL) and their associated cholesterol, known as remnant cholesterol (RC), play a significant role in residual cardiovascular risk. RC encompasses cholesterol present in very-low-density lipoprotein (VLDL), intermediate-density lipoprotein (IDL), and chylomicron (CM) remnants. Measuring RC is especially relevant for patients who continue to experience recurrent cardiovascular events despite receiving intensive lipid-lowering therapy. As highlighted by Bosco and colleagues, integrating RC assessment with inflammatory biomarkers enhances cardiovascular risk prediction beyond what can be achieved with low-density lipoprotein cholesterol (LDL-C) alone, thereby facilitating a more individualized evaluation of residual risk [19]. In September 2021, the European Atherosclerosis Society issued a consensus statement on TRLs and their remnants [20], noting that a wide range of epidemiological and genetic evidence strongly links elevated levels of triglycerides (TG), TRLs, and remnant particles to cardiovascular disease. The statement concluded that TG should be regarded as a causal risk factor for atherosclerotic cardiovascular disease [21] (Table 1).

**Table 1:** Epidemiological and genetic studies on HTG and coronary heart disease

Author	Year published	Sample size	Study setting	Outcome
Nordestgaard, B. G. et al. [22]	2007	13,981	Copenhagen City Heart Study	As triglyceride levels rise, the risks of myocardial infarction, ischemic heart disease, and all-cause mortality significantly increase, with the trends being statistically significant(*P* for trend < 0.001)
Sandeep Bansal et al. [23]	2007	26,509	Women's Health Study	nonfasting triglyceride levels maintained a strong independent relationship with cardiovascular events in fully adjusted models (hazard ratio [95% CI] for increasing tertiles of levels: 1 [reference], 1.44 [0.90–2.29], and 1.98 [1.21–3.25] [*P* = 0.006 for trend])
Nadeem Sarwar et al. [24]	2007	44,237	Reykjavik and EPIC-Norfolk studies	After adjustment for baseline values of several established risk factors, the adjusted odds ratio for coronary heart disease was 1.76 (95% CI, 1.39 to 2.21) in the Reykjavik study and 1.57 (95% CI, 1.10 to 2.24) in the EPIC-Norfolk study in a comparison of individuals in the top third with those in the bottom third of usual log-triglyceride values
Anette Varbo et al. [25]	2013	73,513	Danish descent from Copenhagen	The causal odds ratio for a 1 mmol/l (39 mg/dl) genetic increase of nonfasting remnant cholesterol was 2.8 (95% CI: 1.9 to 4.2), with a corresponding observational hazard ratio of 1.4 (95% CI: 1.3 to 1.5)

The cardiovascular benefit of triglyceride (TG) lowering beyond statin therapy varies across drug classes, highlighting an area of persistent residual risk. Current pharmacological options for hypertriglyceridaemia (HTG) include fibrates, niacin, and omega-3 fatty acids. Among these, only the high-purity omega-3 formulation icosapent ethyl (IPE) has consistently demonstrated cardiovascular event reduction in large-scale outcome trials, positioning it as a targeted therapy for HTG-related residual risk (Table 2).

**Table 2:** Clinical research on drugs for reducing triglycerides

Trail	Year published	Sample size	Groups	Baseline drug	Outcome
FIELD [26]	2005	9795	placebo and fenofibrate	No statin	The primary end point(HR, 0.89, 95% CI, 0.75–1.05; *P* = 0.16)
ACCORD [27]	2010	5518	placebo and fenofibrate	simvastatin	The primary end point(HR, 0.92,95% CI, 0.79–1.08; *P* = 0.32) Subgroup(TG > 204 mg/dL and HDL < 34 mg/dL) (HR 0.73,95% CI, 0.56–0.95; *P* = 0.048)
PROMINENT [28]	2022	10,497	placebo and pemafibrate	With statin	The primary end point (HR, 1.03; 95% CI, 0.91–1.15; *P* = 0.67)
AIM-HIGH [29]	2011	3414	placebo and niacin	Simvastatin plus ezetimibe	The primary end point (HR, 1.02; 95% CI, 0.87–1.21; *P* = 0.79)
JELIS [30]	2007	18,645	High purity EPA + statin and statin only	With statin	The primary end point (HR = 0.81; 95% CI 0.69–0.95; *p* = 0.011)
FISH [31]	2012	201	Placebo and EPA and DHA mixtuer 4 g/d (930mgEPA + 660mgDHA)	-	The primary end point (HR, 0.78; 95% CI, 0.60 to 1.03; *P* = 0.06) Improved cardiovascular event-free survival (HR, 0.43; 95% CI, 0.19 to 0.96; *P* = 0.04])
ORIGIN [32]	2018	12,537	Placebo and n-3 fatty acids	With statin	The primary end point (HR, 0.98;95% CI, 0.87–1.10; *P* = 0.72)
ASCEND [33]	2018	15,480	n-3 fatty acids and olive oil	With statin	The primary end point (RR, 0.97; 95% CI, 0.87–1.08; *P* = 0.55)
VITAL [34]	2019	25,871	Placebo and marine n-3 fatty acids	With statin	The primary end point (HR, 0.92; 95% CI, 0.80–1.06; *P* = 0.24)
REDUCE-IT [35]	2019	8179	Mineral oil and IPE	With statin	The primary end point (HR, 0.75; 95% CI, 0.68 to 0.83; *P* < 0.001)
STRENGTH [36, 37]	2018, 2025	13,078 (1355 Asians)	corn oil and EPA and DHA mixtuer 4 g/d(2200mgEPA + 800mgDHA)	With statin	MACE (0.99 (95% CI 0.90–1.09, *p* = 0.84) Asian subgroup: MACE(HR = 0.81; 95% CI 0.69–0.95; *p* = 0.011) (HR 0.72; 95%CI 0.54–0.96; *p* = 0.03)
OMEMI [38]	2021	1027	corn oil and EPA and DHA mixtuer 1.9 g/d (930mgEPA + 660mgDHA)	With statin	The primary end point (HR, 1.08; 95% CI, 0.82–1.41; *P* = 0.60)
PISCES [39]	2026	1228	EPA and DHA mixtuer 5 g/d (1600mgEPA + 800mgDHA) & corn oil	-	The primary end point (HR 0.57; 95% CI 0.47–0.70; *p* < 0.001)

### Traditional “failed therapies”: fibrates and niacin

The cardiovascular benefits of fibrates (e.g., fenofibrate, bezafibrate, and pemafibrate) remain inconsistent across studies. In their overall study cohorts, major trials including FIELD, ACCORD, BIP, and PROMINENT reported no significant reductions in MACEs [26, 28, 40]. Only subgroup analyses—most notably from FIELD and ACCORD—indicated that patients with very high baseline triglycerides (≥ 200 mg/dL) and/or low HDL-C might derive some benefit [27, 40]. The lack of efficacy observed in PROMINENT could be attributable to intensive background statin therapy combined with pemafibrate-related elevations in LDL-C and apolipoprotein B, which may have counteracted any positive effects from lowering triglyceride-rich lipoproteins [28]. Regarding niacin, despite its broad-spectrum lipid-altering actions, its utility remains limited in statin-treated populations. Contemporary large-scale outcome trials, including AIM-HIGH and HPS2-THRIVE, failed to achieve their primary cardiovascular endpoints [29, 41]. Although a post-hoc analysis of AIM-HIGH suggested a potential niche for niacin in patients presenting with both high triglycerides and low HDL-C [42], this observation lacks confirmation from definitive, large-trial evidence. Both AIM‑HIGH and HPS2‑THRIVE demonstrated that niacin failed to reduce cardiovascular events and was associated with increased adverse events, resulting in premature trial termination.

### A relatively successful therapeutic approach: icosapent ethyl

For patients with hypertriglyceridaemia (HTG), the most convincing evidence of cardiovascular benefit comes from high-purity icosapent ethyl (IPE). Two pivotal studies have established its efficacy. In the JELIS trial, adding high-purity eicosapentaenoic acid (EPA) to statins in a Japanese cohort led to a 19% decrease in major coronary events, and a 53% decrease in those with elevated triglycerides and reduced HDL-C [30]. Later, the REDUCE-IT trial, which had a global scope, reported a 25% relative risk reduction for the primary composite cardiovascular endpoint among high-risk, statin-treated individuals receiving IPE versus placebo [35]. aken together, these trials offer solid evidence that IPE reduces residual cardiovascular risk beyond conventional lipid management. In contrast to fibrates and other traditional triglyceride-lowering medications that yield variable results, high-purity IPE provides the most conclusive support for residual risk reduction in patients already on statin therapy.

Evidence for EPA/DHA mixtures remains inconsistent. The STRENGTH [36] and OMEMI [38] trials found no significant cardiovascular benefit with EPA/DHA mixtures in high-risk populations. In contrast, the FISH trial in chronic haemodialysis patients requiring new graft access reported a > 50% reduction in cardiovascular events with an EPA/DHA mixture (4 g/day) versus placebo (HR 0.43) [31]. Similarly, the PISCES trial in patients on renal replacement therapy demonstrated a 43% reduction in the primary endpoint of cardiovascular death and nonfatal events with an EPA/DHA combination (4 g/day) over a median 3.5-year follow-up (HR 0.57) [39].

The divergent outcomes observed in clinical trials investigating IPE versus EPA/DHA mixtures likely result from several interrelated factors, primarily formulation purity differences, plasma EPA levels, and patient population characteristics. A key differentiating factor is that the REDUCE-IT trial utilised high purity EPA (IPE), whereas other studies used EPA/DHA combinations. Evidence underscores serum EPA concentration as a critical determinant of efficacy, suggesting that benefits may require reaching a specific threshold, a target more reliably achieved with high-dose IPE owing to its pure formulation [43]. The use of a mineral oil placebo in the REDUCE-IT trial, which may exert minor pro-inflammatory and pro-atherogenic effects, has been debated, particularly in contrast to neutral vehicles such as corn oil employed in trials such as STRENGTH. This is illustrated by the divergent hsCRP trends: over two years, levels decreased by 14% in the IPE group but increased by 32% in the placebo group. Although the choice of placebo may have amplified the observed between-group differences, it did not undermine the significant cardiovascular risk reduction demonstrated with IPE therapy. Furthermore, significant population heterogeneity was observed. FISH and PISCES enrolled haemodialysis patients, a group with profound omega-3 deficiency, an inflammatory environment, an exceptionally high CV risk, and a unique pathophysiology, who may be more responsive to supplementation. In contrast, STRENGTH targeted a broader statin-treated population with residual HTG. Notably, a post-hoc analysis of STRENGTH suggested a potential CV benefit in the Asian subgroup (HR 0.72) but not in non-Asian participants, indicating that racial and genetic and baseline metabolic characteristics (e.g. lipid profiles, inflammation, and omega-3 levels) may influence treatment response [37]. Therefore, clinical evidence establishes the unique value of high-purity IPE in reducing the risk of ASCVD, specifically the residual risk associated with elevated TG levels in statin-treated patients. While EPA/DHA mixtures showed benefits in specific high-risk populations with extreme omega-3 deficiency (e.g. haemodialysis patients), their effects were less consistent across broader CV populations than those of IPE. The superior and reproducible CV outcomes of IPE were attributed to its high-purity EPA composition, which effectively lowers TG and achieves the plasma EPA concentrations necessary to exert pleiotropic protective effects. Therefore, in the management of HTG to mitigate the ASCVD risk, high-purity IPE provides the most compelling and validated evidence of significant TG-related cardiovascular risk reduction.

In summary, despite robust genetic and epidemiological links between HTG and ASCVD, the clinical efficacy of TG-lowering therapies remains variable. This discrepancy underscores the critical debate regarding whether HTG is a causative mediator or merely a bystander of atherogenesis. To resolve this, the present review integrates the complex mechanisms linking HTG to cardiovascular risk and provides the first detailed analysis of novel RNA-targeted agents against apolipoprotein C-III (*APOC3*) and angiopoietin-like 3 (ANGPTL3). This synthesis aims​ to refine current risk stratification models and steer the evolution of targeted therapeutic strategies beyond traditional approaches.

## Hypertriglyceridaemia and triglyceride metabolism

To better understand the relationship between HTG and ASCVD, it is important to examine TG metabolism. The 2021 European Atherosclerosis Society consensus document [20] together with the 2023 Chinese guidelines for lipid management [44] recommends the following classification for fasting TG levels: < 1.7 mmol/L as optimal; 1.7–2.3 mmol/L as borderline high; 2.3–5.6 mmol/L as moderately high; and > 5.6 mmol/L as severely high. As there are very few free TG in the circulation, they must combine with proteins and phospholipids to be transported to various tissues in the form of lipoproteins for utilisation or metabolism. The principal triglyceride-rich lipoproteins, CM and VLDL, are also known as TRLs [45]. Lipoprotein lipase acts on CM—TRL particles assembled in the intestine—to release free fatty acids and generate CM remnants. Following this hydrolysis, these smaller remnant particles traverse the space of Disse and are subsequently cleared through multiple hepatic pathways, involving LDL receptors, LRP family members, and heparan sulfate proteoglycans anchored on hepatocyte surfaces.Following hepatic synthesis and secretion, VLDL undergoes hydrolysis of its core TGs by LPL, leading to particle shrinkage and compositional remodelling, which converts VLDL into an intermediate-density lipoprotein (IDL). Subsequently, IDL is either cleared via hepatic LDL receptors or subjected to further hydrolysis by hepatic lipase within the sinusoidal endothelium. This latter step removes residual TGs and phospholipids, culminating in the formation of LDL particles. The major constituents of remnant lipoproteins (RLP) are remnants derived from VLDL and CM. Through the action of plasma cholesteryl ester transfer protein (CETP), TRL remnants exchange lipids with LDL-C and HDL-C: cholesterol esters (CE) from LDL and HDL are transferred to TRLs, while TGs move in the opposite direction [20, 46], This process enables TRL remnants to accumulate more cholesterol than nascent TRLs. Subsequently, free fatty acids (FFAs) released from this process are absorbed by muscle cells and adipocytes. Remnant cholesterol (RC) refers to the cholesterol carried by lipoproteins excluding HDL-C and LDL-C, with the majority residing in VLDL and CM remnants [47]. Although newly synthesised TRLs (such as chylomicrons) are too large to cross the endothelial barrier, their smaller particles, namely the CM and VLDL remnants, can penetrate the intima. These findings have significantly deepened our understanding of the association between HTG and ASCVD.

## Triglyceride-rich lipoproteins promote foam cell formation in plaques

Lipid deposition is the pathological basis of atherosclerosis. LDL is the primary source of cholesterol accumulation within the arterial walls. Mirroring the behaviour of LDL, TRL remnants penetrate the intima via the passive transcytosis of endothelial cells. Their entry into the subendothelial space occurs via two distinct pathways: indirectly mediated by cell membrane surface receptors and directly through the caveolae. Within the subendothelial space, the TRL remnants bind to proteoglycans. This interaction is mediated by the positively charged arginine and lysine residues on apoB in the remnants, which electrostatically interact electrostatically with negatively charged sulfate groups on proteoglycans [48]. However, the transendothelial migration rate of TRL remnants is slower than that of LDL-C, primarily because of their larger size [49]. This increased size also contributes to a higher propensity for endothelial retention than that of LDL-C. Critically, TRL remnants differ fundamentally from LDL-C in their mechanism of macrophage uptake. They can be internalised by macrophages either via specific receptors or directly through scavenger pathways, thereby promoting foam cell formation without prior oxidative modifications. Additionally, the cholesterol content carried by TRL remnants is approximately 5–20 times that of LDL-C because of lipid exchange between TGs in TRLs and CE in LDL through CETP [50, 51]. Furthermore, TRL remnants carry approximately 5–20 times more cholesterol than LDL particles, a disparity primarily attributable to CETP-mediated bidirectional lipid exchange, wherein TGs from TRLs is transferred from LDL for CE [20, 48]. This substantial cholesterol load confers a markedly enhanced atherogenic potential to TRL remnants, primarily by driving foam cell formation. Concomitantly, the lipid exchange process during TRL metabolism generates small dense LDL (sdLDL). The sdLDL particles are inherently susceptible to subendothelial retention and oxidation, thereby promoting foam cell generation.

However, clinical studies have indicated that severe HTG is not associated with atherosclerotic plaque formation, especially in humans with severe HTG secondary to profound LPL deficiency and that the metabolism of VLDL and CM into atherogenic remnants is impaired [48]. These large, non-atherogenic precursor particles exhibit a severely impaired ability to enter the vascular endothelium and thus do not directly contribute to atherosclerotic development. In a seminal 1988 study, Nordestgaard et al. established a rabbit model of severe HTG using alloxan-induced diabetes [52]. They observed significantly reduced atherosclerotic lesion formation in diabetic rabbits with marked HTG levels compared with non-diabetic controls. This apparent protective effect correlated with markedly diminished LPL activity in the diabetic group. This impairment prevents the conversion of VLDL and CM to smaller atherogenic remnant particles. Consequently, large VLDL and CM particles ​ are confined to the plasma and cannot access the subendothelial space, thus precluding plaque formation [53].

## Pro-inflammatory mechanisms of triglyceride-rich lipoproteins

A current method for assessing vascular inflammation involves quantifying 18F-fluorodeoxyglucose (18F-FDG) uptake via positron emission tomography (PET). Data from the Progression of Early Subclinical Atherosclerosis (PESA) study revealed that, following multivariable adjustment, individuals with triglyceride (TG) levels ≥ 150 mg/dL had notably higher arterial 18F-FDG uptake than those with TG < 100 mg/dL (adjusted OR: 2.09; 95% CI: 1.29–3.40; *P* = 0.003). Moreover, a significant positive relationship was observed between serum TG and high-sensitivity C-reactive protein (hs-CRP) levels (Pearson’s r = 0.298; 95% CI: 0.269–0.327; *P* < 0.001) [54]. Recent findings indicate that TRLs and their breakdown products—including RLP and FFAs—engage with endothelial cells, macrophages, and monocytes. This engagement stimulates the release of pro-inflammatory cytokines and adhesion molecules, which in turn aggravates endothelial dysfunction and encourages inflammatory cell migration into the subendothelial space. Collectively, these processes facilitate the formation of atherosclerotic plaques.

### Triglyceride-rich lipoproteins phagocytosis by monocyte and macrophage drives intracellular triglyceride accumulation and promotes inflammatory and foamy cellular phenotype

Recent cell-based studies have shown that elevated VLDL levels lead to TG accumulation in macrophages [55]. At the same time, significant progress has been made in elucidating the mechanisms by which macrophages take up TRLs [56]. Research involving the treatment of primary human macrophages with human plasma-derived VLDL demonstrated that LPL acts as a key bridging factor in the internalization of TRLs. This function is dependent on the C-terminal domain of LPL. VLDL and CM particles are internalized through caveolae-mediated endocytosis. Following internalization, acidic lipases in lysosomes degrade these particles, liberating fatty acids that are then transported to the endoplasmic reticulum (ER) by STARD3, a lipid transfer protein [55]. Once in the ER, these fatty acids are re-esterified into TGs for cytosolic storage. Subsequently, TGs can be exported to the extracellular space via NPC1-dependent pathways. The buildup of intracellular TG in macrophages increases CD36 expression, a major scavenger receptor responsible for oxLDL uptake, which in turn hastens foam cell formation [55]. Additionally, experiments on THP-1 monocyte-derived macrophages exposed to non-esterified fatty acids (NEFAs) showed a dose-dependent promotion of foam cell formation. The underlying mechanism involves NEFA-mediated enhancement of CD36 activity, which boosts LDL-C uptake and accelerates the transformation into foam cells [57].

TRL metabolites activate macrophage pattern-recognition receptors (PRRs), triggering downstream PKC-MAPK-RAS signaling cascades that promote secretion of pro-inflammatory cytokines including IL-1β and TNF-α. Conversely, oxLDL upregulates the pro-inflammatory TREM-1 pathway while suppressing the atheroprotective, anti-phagocytic functions of TREM-2, thereby disrupting macrophage homeostasis and amplifying vascular wall inflammation. TRLs also induce pro-inflammatory cytokine expression and drive macrophage polarization toward the pro-inflammatory M1-like phenotype via multiple signaling pathways.​

**Fig. 1:**
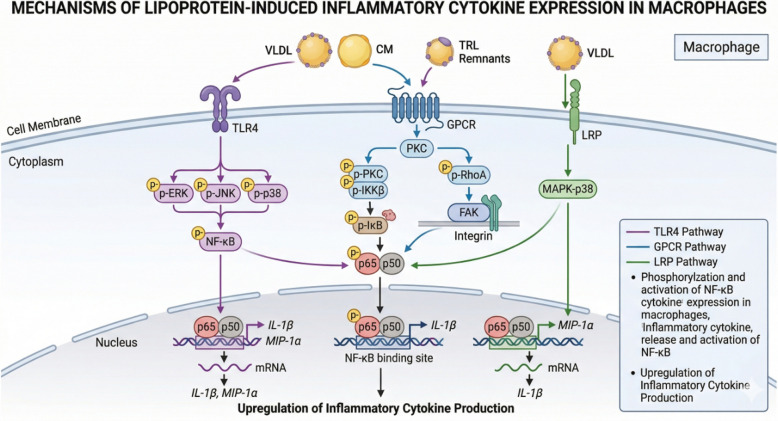
Macrophage inflammation pathway map. TRL metabolites signal through macrophage pattern-recognition receptors (GPCRs/TLR4/LRPs), activating PKC-MAPK-RAS signaling axes to drive IL-1β/TNF-α secretion. TRLs promote the inflammatory cytokines productions in macrophages through multiple signaling pathways and drive phenotypic polarization toward the pro-inflammatory M1-like state

Mechanistically, VLDL, chylomicrons (CM), and remnant lipoproteins (RLP) activate Toll-like receptor 4 (TLR4) on the cell membrane, triggering the MAPK–ERK1/2/JNK/p38–NF-κB axis to upregulate IL-1β and macrophage inflammatory protein-1α (MIP-1α) expression [58]. Concurrently, they engage G protein-coupled receptors (GPCRs) to activate the PKC–IκB or PKC–RhoA–FAK interferon signaling pathways, which act on NF-κB-dependent nuclear transcription to promote IL-1β expression. VLDL and their remnants also signal via LDL receptor-related proteins (LRPs) and p38 MAPK, further enhancing IL-1β synthesis [59]. Importantly, foam cell studies showed that pharmacological inhibition of TG synthesis reduced PGE₂ biosynthesis and attenuated downstream IL-1β/IL-6 production [60]. Thus, TG stored in foam cells promotes inflammatory factor expression by enhancing PGE₂ synthesis (Fig. 1).

**Fig. 2:**
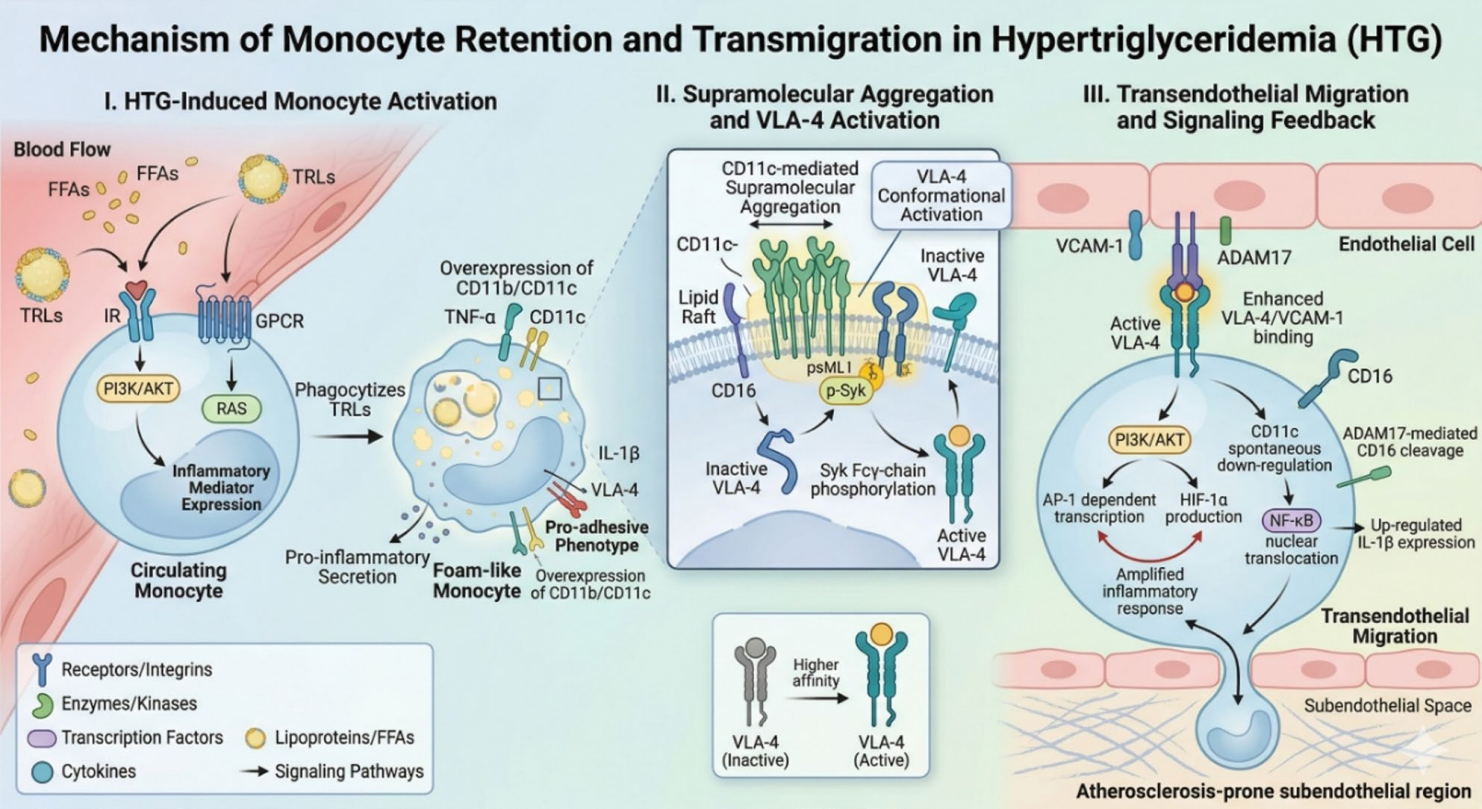
Hypertriglyceridemia -Driven Monocyte inflammation polarization. Free fatty acids (FFAs) activate insulin receptor (IR) and G protein-coupled receptor 120 (GPR120) on monocytes, driving inflammatory mediator expression via PI3K/AKT and RAS signaling cascades. A HTG environment increases the CD11c^+^ phenotype of monocytes, promote the aggregation of CD16, p-Syk, and VLA-4 in stable complexes within membrane lipid rafts as well as the phosphorylation of Syk Fc-γ chain, thereby inducing VLA-4 activation, facilitating the connection between VLA-4 and VCAM-1, and stimulating the migration of monocytes to endothelial cells. Furthermore, VLA-4 ligation amplifies inflammatory responses through PI3K/AKT-dependent AP-1 transcriptional activation and HIF-1α stabilization

Macrophages in the vascular wall originate from resident tissue macrophages or monocytes differentiated from circulating precursors. In an HTG environment, circulating monocytes phagocytose TRLs and form foam. Subsequently, these cells acquire a pro-adhesive phenotype via CD11b/CD11c overexpression and pro-inflammatory secretory profile (TNF-α/IL-1β), facilitating integrin-mediated endothelial arrest and subsequent diapedesis into atherosclerosis-prone subendothelial niches [56]. An *in vitro* study revealed that apolipoprotein E on the surface of TRLs interacts with monocytes expressing LRP-1 and VLDLR, thereby mediating the internalisation of TRLs in monocytes and promoting cellular lipid aggregation [56]. Mice fed a Western high-fat diet exhibit a significant increase in circulating foamy monocytes compared to controls fed a normal diet [61]. This expansion was primarily driven by selective enrichment of a CD11c⁺CD36⁺ monocyte subpopulation. Compared to non-foamy monocytes, these cells showed higher surface expression of CD11c and CD36, along with increased production of TNF-α and IL-1β. CD11c-mediated lipid raft clustering recruits CD16, p-Syk, and VLA-4, triggering Fcγ chain phosphorylation and conformational activation of VLA-4. Activated VLA-4 binds VCAM-1 with enhanced affinity, driving monocyte diapedesis across the endothelium. VLA-4 activation also induces AP-1-dependent transcription via the PI3K/AKT pathway and stimulates HIF-1α production, amplifying inflammatory responses. Following VLA-4/VCAM-1 ligation, CD11c activity is downregulated, while ADAM17-mediated CD16 cleavage occurs alongside NF-κB nuclear translocation, which upregulates IL-1β expression. This cascade reprograms monocytes toward a proinflammatory phenotype [62] (Fig. 2). Circulating foamy monocytes infiltrate the arterial wall and peripheral tissues, differentiating into giant foam cells with enhanced phagocytic capacity [63]. Although large TRLs cannot directly cross the endothelium, they access the subendothelial space via engulfment by circulating monocytes acting as "Trojan horse" vectors.

FFAs activate the insulin receptor (IR) and G protein-coupled receptor 120 (GPR120) on monocytes, driving the expression of inflammatory mediators via the PI3K/AKT and RAS signalling cascades. A HTG environment increases the CD11c^+^ phenotype of monocytes, promotes the aggregation of CD16, p-Syk, and VLA-4 in stable complexes within membrane lipid rafts as well as the phosphorylation of the Syk Fc-γ chain by Syk, thereby inducing VLA-4 activation, facilitating the connection between VLA-4 and VCAM-1, and stimulating the migration of monocytes to endothelial cells. Furthermore, VLA-4 ligation amplifies inflammatory responses through PI3K/AKT-dependent AP-1 transcriptional activation and HIF-1α stabilisation. Additionally, CETP-driven lipid reshuffling between TRLs and HDL remodels HDL into pro-inflammatory particles with diminished antioxidant capacity and impaired cholesterol efflux function, ultimately subverting HDL's vasoprotective role and fuelling vascular inflammation [56].

### Triglyceride-rich lipoproteins and their metabolites promote arterial wall inflammatory responses and cellular ageing

#### Triglyceride-rich lipoproteins promote endothelial cell inflammatory responses and ageing

In vitro experiments have demonstrated that remnant lipoproteins (RLP) dose-dependently elevate the mRNA expression of ICAM-1, VCAM-1, and tissue factor in human endothelial cells. Consequently, the upregulation of ICAM-1 and VCAM-1 promotes the adhesion of leukocytes and neutrophils, highlighting the pro-inflammatory role of RLP [49].

During lipolysis, catalysed by LPL on vascular surfaces, TRLs release abundant FFAs. These FFAs bind to integrin receptors on the endothelial cell membranes and activate PTEN. This subsequently suppresses the PI3K-PIP3-AKT signalling pathway, thus inhibiting eNOS and reducing the production of the anti-inflammatory mediator NO. Concurrently, this cascade promotes inflammation and stimulates ROS generation via the NADPH oxidase and Cytochrome P450 systems in endothelial cells.

**Fig. 3:**
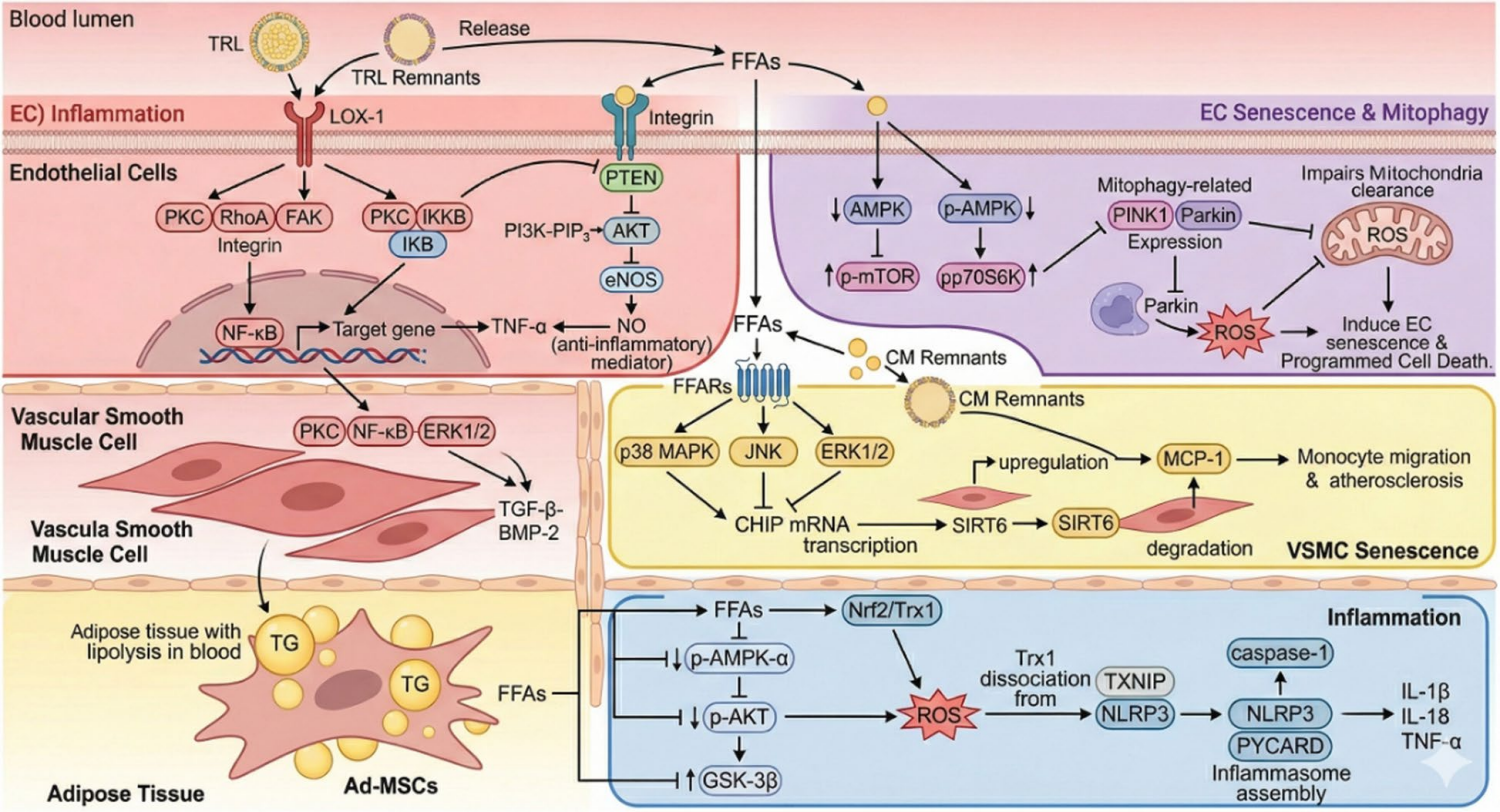
Pathway map of TRL and its metabolites on endothelial cell, VSMC, and fat tissue. TRLs and their metabolites affect endothelial cells via LOX-1 and integrin receptors. This signaling promotes TNF-α production while inhibiting NO production. FFA-mediated inhibition of mTOR. This inhibition suppresses downstream targets p-mTOR and p70S6K, leading to reduced expression of the mitophagy-related proteins PINK1 and Parkin. Consequently, Parkin enrichment into mitochondria is impaired, preventing the timely clearance of damaged mitochondria. This results in excessive ROS production and promotes endothelial cell aging. FFAs act on the FFAR (Free Fatty Acid Receptor) on vascular smooth muscle cells (VSMCs) and activating the MAPK/p38 signaling pathway. This process promotes the expression of an aging phenotype in VSMCs and suppresses expression of the anti-aging factor SIRT6. FFAs inhibit the AMPK pathway in adipocytes. This inhibition activates GSK-3β and disrupts the Nrf2/Trx1 antioxidant axis, leading to increased ROS levels. The disruption causes Trx1 to dissociate from TXNIP, thereby enhancing the TXNIP-NLRP3 interaction. This interaction stimulates the NLRP3/PYCARD/caspase-1 inflammasome pathway, resulting in the production and secretion of the inflammatory cytokines IL-1β, IL-18, and TNF-α

Endothelial dysfunction resulting from NO/ROS imbalance promotes monocyte/macrophage infiltration and foam cell formation [59, 64]. RLP triggers LOX-1 activation in endothelial cells, thereby enhancing NF-κB-mediated transcription and TNF-α synthesis. The resulting upregulation of adhesion molecules (VCAM-1, ICAM-1, E-selectin) and MCP-1 stimulates superoxide production via NAD(P)H oxidase, as well as cytokine secretion, ultimately compromising endothelial cell survival [56, 65] (Fig. 3).

Recent studies have demonstrated that TRL metabolites promote cellular senescence in atherosclerosis [66, 67]. Senescent cells exhibit characteristic phenotypic changes and impaired organelle function, which further reduce vascular elasticity. The accumulation of large numbers of senescent cells damages the arterial endothelium and accelerates atherosclerotic lesion formation [68]. Evidence indicates that LDL and its modified forms drive atherosclerosis by inducing senescence in vascular cell senescence [69]. Numerous studies have confirmed that the lipolytic products of TRLs accelerate cellular senescence [66, 67], thereby contributing to atherosclerotic formation.

In a previous study, vascular endothelial cells treated with RLP from healthy participants after a high-fat diet showed significantly elevated peroxynitrite (ONOO⁻) levels—a product of NO and superoxide—in senescent cells. Elevated RLP increased ROS production, inhibited AKT and telomerase reverse transcriptase phosphorylation, and suppressed telomerase activity. Under high-glucose conditions, particularly with diabetes, PGC-1α upregulated the SIRT1/p53/p21 pathway, accelerating endothelial senescence [70]. Endothelial cells are critical for maintaining vascular structural integrity and homeostasis [71]. Senescent endothelial cells compromise vascular integrity and permeability, allowing plasma LDL, TRLs, and other atherogenic lipoproteins to enter the subendothelial space. Moreover, they produce more senescence-associated secretory phenotype (SASP) factors, which recruit and activate macrophages and monocytes in the subendothelial layer, promoting oxidized LDL phagocytosis. Collectively, these processes drive foam cell formation and accelerate atherosclerosis [72, 73].

Palmitic acid is the primary form of FFAs in the human body. An *in vitro* cellular study [66] demonstrated that the treatment of human aortic endothelial cells with palmitic acid significantly reduced AMPK and phosphorylated AMPK (p-AMPK) protein levels. Conversely, its downstream targets phosphorylated mTOR (p-mTOR) and phosphorylated p70S6K (pp70S6K) exhibited increased activation. This molecular dysregulation reduces the expression of PINK1 and Parkin proteins, which are critical for mitophagy, inhibits Parkin translocation to the mitochondria, and impairs the timely clearance of damaged mitochondria, ultimately resulting in excessive ROS generation (Fig. 3). Elevated ROS levels induce programmed endothelial cell death [74]. Additionally, palmitic acid was found to decrease the LC3B-II/β-actin expression ratio [66]. Because LC3B-II is a key marker and initiator of autophagy, its reduction impairs the autophagic pathway, leading to the accumulation of damaged cellular components.

#### Triglyceride-rich lipoproteins metabolites promote the senescence of vascular smooth muscle cells

Senescent vascular smooth muscle cells (VSMCs) adopt a prosecretory phenotype characterized by elevated MMP production and secretion. This enhanced MMP activity degrades elastic fibers, thins the fibrous cap, increases plaque vulnerability, and ultimately leads to plaque rupture and thrombosis [67]. SIRT6 (Sirtuin 6), a nuclear deacetylase, counteracts VSMC senescence by preserving telomeres through deacetylation of telomeric chromatin. In vitro experiments showed that treating human VSMCs with palmitic acid reduced SIRT6 protein levels. Mechanistically, palmitic acid activates the p38 MAPK and JNK pathways while suppressing the ERK1/2 pathway, which lowers CHIP mRNA transcription and accelerates SIRT6 degradation. Additionally, palmitic acid drives phosphorylation within the PKC/NF-κB–ERK1/2 axis, increasing production of TGF-β and BMP-2—both markers of VSMC senescence. These changes collectively promote VSMC calcification and undermine plaque stability [70] (Fig. 3). Notably, unlike their senescence-inducing effect on endothelial cells, TRLs accelerate atherosclerosis primarily by provoking inflammatory responses in VSMCs. Specifically, chylomicron remnants substantially upregulate MCP-1 expression in VSMCs via a p38 MAPK-dependent mechanism. MCP-1 is a key chemokine that recruits monocytes and plays a pivotal role in atherogenesis [75]. Furthermore, MCP-1 induces early growth response factor-1 (Egr-1) expression through the ERK/MAPK pathway (Fig. 3). Egr-1, a zinc-finger transcription factor, activates pro-atherogenic genes [76].

### HTG aggravates adipose tissue inflammation and endocrine dysfunction

FFAs generated through lipolysis are taken up by adipose tissue and stored as TGs within adipose tissue-derived stem cells. A murine study found that both IL-1β mRNA expression and protein levels were upregulated in the adipose tissue of wild-type mice fed a high-fat diet. Furthermore, treatment of adipose tissue with the NLRP3 inflammasome inhibitor MCC950 resulted in reduced IL-1β levels [77]. Moreover, FFAs specifically inhibit the AMPK pathway in adipocytes, thereby reducing phosphorylation of the AMPK-alpha subunit. This inhibition consequently decreases AKT phosphorylation, leading to activation of GSK-3β. Suppression of the Nrf2/Trx1 antioxidant axis by FFAs increased ROS levels. Elevated ROS levels promote the dissociation of Trx1 from TXNIP, enhancing the TXNIP-NLRP3 interaction. This interaction stimulates the NLRP3-PYCARD-caspase-1 pathway, triggering inflammasome assembly and subsequent production of the inflammatory cytokine IL-1β [77]. The release of IL-18 and IL-1β then signals through MyD88 to induce TNF-α expression, ultimately driving inflammation [77–79] (Fig. 3).

## Promotion of thrombus formation

Thrombosis, secondary to atherosclerotic plaque rupture, is the primary pathological mechanism underlying acute ischaemic vascular events. Fundamental mechanisms driving thrombosis include vascular endothelial injury, alterations in blood flow, and changes in blood composition. Emerging evidence indicates that HTG contributes to thrombotic events by disrupting the balance between anti- and prothrombotic factors.

### HTG increases blood viscosity

Although normal blood vessel walls possess inherent anti-thrombotic properties, vascular injury disrupts this protective function and promotes thrombus formation. Evidence from clinical trials shows that high plasma levels of VLDL remnants contribute to the onset of endothelial dysfunction. In particular, excess VLDL remnants bind to and lower circulating levels of free tissue factor pathway inhibitor (TFPI), reducing its endothelial localization. This TFPI deficit, along with the direct actions of VLDL remnants, fosters endothelial injury and increases platelet aggregation [80]. Hemodynamic factors, including blood flow velocity and viscosity, also significantly influence thrombus formation. Plasma viscosity, together with erythrocyte traits such as concentration, volume, deformability, and aggregation, primarily determines blood viscosity [81]. Robert et al. [82] studied 257 consecutive individuals (169 White, 47 Black, 23 Hispanic, and 11 from other racial/ethnic groups) undergoing cardiovascular risk assessment at the Rush Heart Institute's Center for Preventive Cardiology. After controlling for viscosity-related confounders including fibrinogen, total serum protein, HDL-C, and hematocrit, plasma TG levels remained significantly associated with plasma viscosity (*P* < 0.0005), suggesting that elevated TG directly raise plasma viscosity. In vitro experiments have also demonstrated that CM, VLDL, and LDL cause dose-dependent exponential rises in plasma viscosity, with the magnitude of these effects correlating positively with lipoprotein particle size [83]. Another key determinant of blood viscosity is erythrocyte characteristics. Research using LPL-deficient (LPL⁻/⁻) mouse models reveals that severe HTG induces red blood cell abnormalities, such as decreased deformability, altered electrophoretic mobility, reduced membrane fluidity, and increased osmotic fragility. A rise in plasma viscosity impairs microvascular perfusion, intensifies endothelial shear stress injury, and promotes plasma protein deposition on narrowed vascular surfaces. Together, these hemodynamic changes create a prothrombotic environment during disease progression [59].

### HTG impairs endogenous antithrombotic mechanisms

Under physiological conditions, the body maintains haemostasis through a sophisticated regulatory system involving vascular integrity, platelet function, coagulation cascades, and fibrinolysis. A dynamic equilibrium between the procoagulant and fibrinolytic activities preserves normal blood fluidity. Pathological thrombus formation occurs when coagulation becomes hyperactivated concurrently with fibrinolysis suppression, establishing a hypercoagulable state that predisposes the patient to thrombosis. A clinical study comprising 18 patients with HTG and 11 normolipidaemic controls preliminarily assessed 24-h variations in plasma TG and fibrinolytic activity. Measurements collected at ten distinct time points revealed that patients with HTG had substantially lower fibrinolytic activity and higher coagulation Factor X levels compared to control subjects. After dietary adjustments and pharmacological treatment, this patient group showed marked reductions in TG levels alongside improved fibrinolytic function and elevated concentrations of both Factor X and Factor Ⅶ [84]. Phosphatidylethanolamine, which is enriched in TRLs, serves as a strong activator of coagulation Factor Ⅶ, thereby triggering the intrinsic coagulation cascade. In vitro experiments further showed that VLDL promotes the release of plasminogen activator inhibitor-1 (PAI-1) from human umbilical vein endothelial cells and HepG2 cells through binding to the LDL receptor (LDLR) [85, 86]. Similarly, chylomicron remnants enhance endothelial PAI-1 production via MAPK signalling and redox activation [87]. Collectively, these findings establish that HTG promotes systemic hypercoagulability by augmenting procoagulant factors, suppressing fibrinolysis through PAI-1 upregulation, and disrupting the coagulation-fibrinolysis equilibrium.

Platelet hyperactivity is also a determinant of hypercoagulability in HTG patients. Clinical studies have demonstrated enhanced platelet activation in patients with primary HTG, potentially through VLDL-mediated platelet stimulation. The positively charged lysine/arginine residues on ApoC-III located on the surface of TRLs neutralise the negative charges on the platelet surface, alter membrane lipid composition, and induce oxidative stress, thereby collectively promoting platelet hyperviscosity and aggregation [80]. By suppressing fibrinolytic activity, facilitating prothrombin complex assembly, elevating PAI-1 and its antigen levels, and enhancing tissue factor expression in endothelial cells, TRLs and their biologically active lipid components trigger platelet aggregation and thrombogenesis. [49, 59].

Thus, HTG promotes thrombogenesis through multiple pathophysiological mechanisms including vascular endothelial injury, increased blood viscosity, systemic hypercoagulability, suppression of fibrinolytic activity, and enhanced platelet hyperaggregation.

## New drugs in treating HTG

### Apolipoprotein C-III inhibitors

ApoCIII plays a central role in controlling TG metabolism. It suppresses LPL activity, which slows the breakdown of TGs within TRLs, and also hampers the hepatic removal of TRL remnants by blocking their interaction with receptors including LDLR and LRP1 [88, 89]. Human genetic evidence has established that individuals carrying loss-of-function mutations in APOC3 have a reduced risk of coronary heart disease [90]. Consequently, targeting apoCIII pharmacologically has emerged as a promising therapeutic direction, with plozasiran and olezarsen as the frontrunners. Plozasiran is an siRNA-based agent. In the Phase III PALISADE trial, a 25 mg dose administered subcutaneously every three months produced an 80% drop in TG levels from baseline after a median of 10 months of follow-up. The incidence of pancreatitis was also significantly lower in the plozasiran group than in the placebo group (OR 0.17; 95% CI 0.03–0.94; *P* = 0.03) [91]. Olezarsen, an antisense oligonucleotide designed to target APOC3 mRNA, exhibited robust TG-lowering efficacy in the large Phase III ESSENCE-TIMI 73b trial [92]. Nevertheless, it remains unproven whether inhibiting apoCIII translates into improved cardiovascular outcomes, and dedicated long-term trials are necessary to address this question. Current clinical guidelines recommend apoCIII inhibitors only for select patient populations, such as those with familial chylomicronemia syndrome [93] Widespread use for ASCVD risk reduction must await positive results from outcome trials.

### Angiopoietin like protein 3 inhibitors

Derived from the liver, angiopoietin-like protein 3 (ANGPTL3) functions as a major suppressor of lipid metabolism. Emerging evidence suggests that the lipid-modifying effects of ANGPTL3 blockade occur via two distinct but complementary routes—one independent of the LDL receptor (primary) and another dependent on it (secondary).

#### Low density lipoprotein receptor-independent pathway: very low density lipoprotein remodeling and clearance mediated by endothelial lipase

In LDLR knockout mice, ANGPTL3 inhibition effectively lowers LDL-C levels, demonstrating that this pathway is independent of the LDLR [94]. This mechanism represents the core pathway through which ANGPTL3 inhibitors lower LDL-C levels, accounting for their efficacy, even in patients with complete LDLR deficiency [95]. Under physiological conditions, ANGPTL3 suppresses EL activity, which impairs VLDL lipolysis and metabolism, resulting in the extensive conversion of VLDL to LDL. Following ANGPTL3 inhibition, EL activity is restored. EL-mediated lipolysis and structural remodelling reduce VLDL particle size and alter its core lipid composition, resulting in the rapid clearance of remnant particles by the liver, thereby reducing the conversion of LDL precursors into LDL and decreasing plasma LDL-C levels [96, 97]. Furthermore, EL directly facilitates hepatic LDL uptake. Upon activation, EL directly mediates LDL clearance from the liver via the heparan sulfate proteoglycan (HSPG) pathway [98].

#### Low density lipoprotein receptor-dependent pathway: clearance mediated by the low density lipoprotein receptor

When ANGPTL3 is inhibited, the surface of the VLDL remnants generated through EL-mediated remodelling becomes enriched with ApoE. These remnant particles are then efficiently recognised, taken up, and cleared by the liver via LDL receptors and related proteins (such as LRP1). Therefore, this pathway depends on the functional integrity of the LDL receptors. In patients receiving statins and PCSK9 inhibitor therapy, LDL expression is upregulated, which amplifies the contribution of the LDL receptor-dependent pathway, thereby producing a synergistic lipid-lowering effect with ANGPTL3 inhibitors [89]. ANGPTL3 inhibitors lower LDL-C levels via a dual-mechanism approach, which is a unique advantage that distinguishes them from conventional lipid-lowering agents.

#### Representative drugs and emerging frontiers

Multiple therapeutic platforms are available for inhibiting ANGPTL3, ranging from monoclonal antibodies to RNA-based agents and in vivo gene editing. The OLE ELIPSE trial evaluated evinacumab—a fully human monoclonal antibody targeting ANGPTL3—in patients with homozygous familial hypercholesterolemia (HoFH). Results showed a 24% relative risk reduction in MACE with evinacumab versus placebo (*P* = 0.0267) [99]. Although this finding offers preliminary support for cardiovascular benefit, it should be interpreted with caution, as the study population was limited to a very-high-risk cohort.

Vupanorsor, a GalNAc-conjugated antisense oligonucleotide that targets ANGPTL3 mRNA, exemplifies the difficulties inherent in RNA-based therapeutic approaches. In the TRANSLATE-TIMI 70 trial, it produced dose-dependent reductions in TG (up to 56.8%) and VLDL-C (60–80%). However, its development was subsequently discontinued due to an unfavorable balance between benefits and risks. Notable adverse effects, such as elevated liver enzymes and dose-dependent decreases in platelet counts, underscore the typically narrow therapeutic window of antisense-based platforms [100, 101].

The most transformative frontier lies in vivo CRISPR base editing. VERVE-201 utilizes an adenine base editor delivered via GalNAc ligands to specifically target the asialoglycoprotein receptor on hepatocytes, enabling permanent inactivation of the ANGPTL3 gene without inducing DNA double-strand breaks. Preclinical studies in LDL receptor-deficient non-human primates demonstrated profound and durable efficacy, with a 46% reduction in LDL-C and 54% reduction in TG [102]. Currently entering Phase 1b trials (NCT06451770), VERVE-201 represents a paradigm shift toward single-dose, curative therapies [103].

Notably, current guidelines confine the approved ANGPTL3 inhibitor (evinacumab) to HoFH treatment [93], underscoring that the broader cardiovascular benefits of ANGPTL3 inhibition remain to be established in long-term outcome trials.

## Strengths and limitations

### Strengths

This review has several strengths that extend beyond the existing literature. To the best of our knowledge, this is the first comprehensive review to systematically bridge the multifaceted pathophysiological mechanisms of HTG with the rapidly evolving landscape of RNA-targeted therapeutics. ​ Unlike previous narratives that primarily focused on statin therapy or general lipid management, we uniquely delineated how TRL remnants drive atherosclerosis through five distinct pathways (lipid deposition, inflammation, senescence, thrombosis, and endothelial dysfunction) while simultaneously detailing novel pharmacological strategies targeting the apoCIII and ANGPTL3 pathways. Second, the inclusion of up-to-date clinical trial data (2023–2026) provides a timely synthesis of emerging therapies. By integrating recent results from trials involving plozasiran (ARO-apoCIII), olezarsen, and vupanorsen, we offer a nuanced analysis of the differential efficacy and safety profiles of these agents, thereby clarifying their potential roles in future clinical practice. Third, we propose a mechanism-based framework for individualized treatment selection.​ This conceptual model bridges the gap between a complex pathophysiological understanding and clinical decision-making, offering practical value for clinicians managing patients with HTG. This review focuses on precision medicine approaches for lipid management by linking specific molecular defects (e.g. apoCIII excess vs. ANGPTL3 dysregulation) to tailored therapeutic targets.

### Limitations

Several limitations should be acknowledged. First, data for novel gene-silencing agents are largely limited to early-phase trials with small cohorts and short follow-up, lacking definitive long-term outcome data. Second, our proposed mechanistic framework remains preliminary and requires prospective validation. Third, the fast-paced nature of drug development may result in the omission of very recent data despite rigorous searching. Finally, the narrative format of this review, unlike a systematic meta-analysis, carries an inherent risk of selection bias.

## Conclusions

Current findings reveal that even when statins—either alone or combined with other lipid-lowering therapies—lower LDL-C to very low levels, a considerable residual risk of ASCVD persists. Data from epidemiological, genetic, and cohort studies have consistently shown a positive association between HTG and ASCVD risk, pointing to elevated TGs as a key contributor to this residual risk. This view is reinforced by evidence that TG levels measured during statin therapy independently predict cardiovascular events. Nevertheless, a paradox emerges: clinical trials evaluating conventional TG-lowering agents—such as fibrates, niacin, and select fish oil preparations—have generally failed to demonstrate meaningful reductions in major ASCVD events, casting doubt on whether HTG plays a causal role. This review systematically delineates the direct mechanistic links through which TRLs and their cholesterol-enriched remnants drive atherothrombosis. The evidence synthesised demonstrates that TRLs contribute to ASCVD pathogenesis through multiple distinct and interconnected pathways: (1) intimal retention and direct foam cell formation; (2) induction of oxidative stress, phenotypic drift, and endothelial adhesion in monocytes/macrophages via intracellular lipid storage; (3) promotion of a pro-inflammatory state through activation of innate immune receptors; (4) induction of endothelial and VSMC dysfunction, senescence, and impaired autophagy; and (5) creation of a systemic prothrombotic state. This comprehensive pathophysiological framework robustly establishes HTG not as a mere biomarker, but as a direct pathogenic driver of ASCVD.

Critically, this mechanistic understanding was validated by the evidence of clinical outcomes. Among TG-lowering therapies, high-purity IPE provides the most robust and reproducible evidence for reducing residual CV risk. Landmark trials, including JELIS in a Japanese population and the global REDUCE-IT trial in high-risk, statin-treated patients, have definitively demonstrated that IPE significantly reduces major adverse CV events, providing a validated therapeutic strategy to mitigate the CV risk associated with HTG. Consequently, effective management of HTG is imperative for comprehensive ASCVD risk reduction. Beyond established agents, such as IPE, novel therapeutic strategies targeting key regulators of TRL metabolism, such as apoCIII and ANGPTL3 inhibitors, are emerging. Their ultimate role in reducing CV events requires validation in dedicated outcome trials.

Building on these conclusions, this review has several direct implications for clinical practice. First, it supports a paradigm shift from viewing HTG merely as a biomarker for recognising remnant cholesterol as a primary therapeutic target, prompting clinicians to prioritise TRL-C lowering in high-risk patients. Second, the emergence of RNA-targeted therapies (e.g. apoCIII/ANGPTL3 inhibitors) offers crucial alternatives for patients with severe HTG refractory to conventional treatments. Third, we proposed a mechanism-based framework to advance precision medicine by matching specific phenotypes (e.g. FCS vs. multifactorial HTG) to tailored therapies. Finally, these insights reinforce the utility of nonfasting TG and remnant cholesterol assessments​ to capture residual risk. Future research should focus on four key areas: large‑scale outcome trials to confirm if apoCIII/ANGPTL3 inhibition reduces hard endpoints; head‑to‑head comparisons with existing therapies (e.g., IPE, pemafibrate) to define relative efficacy; rational combination strategies, such as adding apoCIII inhibitors to statins; and cost‑effectiveness analyses to guide clinical adoption post‑approval.

## Supplementary Information

Supplementary Material 1.

Supplementary Material 2.

## Data Availability

No datasets were generated or analysed during the current study.

## Abbreviations

^18^F-FDG: 18F-fluorodeoxyglucose
ACCORD: Action to Control Cardiovascular Risk in Diabetes
AIM-HIGH: Atherothrombosis Intervention in Metabolic Syndrome with Low HDL/High Triglyceride and Impact on Global Health Outcomes
ApoCIII/APOC3: Apolipoprotein C-III
ASCVD: Atherosclerotic cardiovascular disease
CI: Confidence interval
CM: Chylomicron
EPA: Eicosapentaenoic acid
FFAs: Free fatty acids
GPCR: G protein-coupled receptor
HDL-C: High-density lipoprotein cholesterol
HPS2-THRIVE: Heart Protection Study 2-Treatment of HDL to Reduce the Incidence of Vascular Events
HR: Hazard ratio
JELIS: Japan EPA Lipid Intervention study
LDL-C: Low-density lipoprotein cholesterol
LPL: Lipoprotein lipase
LRPs: LDL receptor-related proteins
MCP-1: Monocyte chemoattractant protein-1
NO: Nitric oxide
PCSK9: Proprotein convertase subtilisin/kexin type 9
PROMINENT: Pemafibrate to Reduce Cardiovascular Outcomes by Reducing Triglycerides in Diabetic Patients
PROVE IT-TIMI22: Pravastatin or Atorvastatin Evaluation and Infection Therapy–Thrombolysis in Myocardial Infarction 22 trial
REDUCE-IT: Reduction of Cardiovascular Events with Icosapent Ethyl–Intervention Trial
RLP: Residual lipoprotein
TLR4: Toll-like receptor 4
TG: Triglycerides
VLDL: Very-low-density lipoprotein
